# Lack of Awareness of the Impact of Improperly Disposed Of Medications and Associated Factors: A Cross-Sectional Survey in Indonesian Households

**DOI:** 10.3389/fphar.2021.630434

**Published:** 2021-04-26

**Authors:** Sofa D. Alfian, Widya N. Insani, Eli Halimah, Nabilla A. Qonita, Siti S. Jannah, Nisa M. Nuraliyah, Woro Supadmi, Vesara A. Gatera, Rizky Abdulah

**Affiliations:** ^1^ Department of Pharmacology and Clinical Pharmacy, Universitas Padjadjaran, Jatinangor, Indonesia; ^2^ Center of Excellence in Higher Education for Pharmaceutical Care Innovation, Universitas Padjadjaran, Jatinangor, Indonesia

**Keywords:** unused medications, awareness, medication disposal practices, pharmaceutical waste, risk factors

## Abstract

**Introduction:** Disposal of unused medications through environmentally unsafe routes is common in Indonesia. The lack of awareness of the impact of improperly disposed of medications is a significant contributing factor. The objectives of this study were to identify factors associated with lack of awareness of the impact of improperly disposed of unused medications and to assess the associations of awareness with medication disposal practices among the general population in Indonesia.

**Patients and methods:** An observational cross-sectional survey was conducted using nonprobability sampling in Bandung, Indonesia, from November 2017 to January 2018 among respondents who were older than 18 years, had used any medication in the past, were literate, and had signed an informed consent document. Disposal practices and awareness regarding the impact of improperly disposed of unused medications were collected using an online- and a paper-based pre-validated questionnaire. The paper-based questionnaires were distributed to respondents in public places such as city center, markets, and religious places. Binary logistic regression was performed to assess associations of sociodemographic and other related factors with a lack of awareness. Odds ratios (ORs) with 95% confidence intervals (CIs) are reported.

**Results:** Of 497 participating respondents, 433 and 64 respondents filled an online- or a paper-based questionnaire, respectively. Most respondents were female, aged between 18 and 30 years, and students/university students. Of 497 respondents, more than half (53.1%) were not aware that improper medication disposal could harm the environment and population health. Most respondents (79.5%) had never received information about proper medication disposal practices. The education level, the number of stored medications at home, and previous education about medication disposal practices were significantly associated with awareness of proper practices. In the multivariate analysis, only those with previous education about medication disposal practices were less likely to report a lack of awareness (OR: 0.043; 95% CI: 0.02–0.09). Respondents with a lack of awareness tended to dispose of their unused medications in the garbage or shared them with friends or relatives.

**Conclusion:** There is a clear need to increase awareness of the importance of proper medication disposal practices, in particular among the student population of Bandung city, Indonesia. Healthcare providers can play an important role by educating this specific population on the proper disposal of unused medications.

## Introduction

The prevalence of unused medications in general population households has substantially increased recently, which can lead to medication wastage (Makki et al., 2019). The prevalence of unused medications was 89.3% (N = 337) and 85.2% (N = 263) in Saudi Arabia and Ethiopia, respectively (Kassahun and Tesfaye, 2020; Wajid et al., 2020). Furthermore, 2 of 3 prescribed medications were reported unused in households in the United States (Law et al., 2015). Unused medications refer to medications that are deteriorated, discontinued, expired, or unintended for any future use because of adverse effects, nonadherence, alteration of dosage, or improved condition (Seehusen and Edwards, 2006; Ruhoy and Daughton, 2008; Law et al., 2015). In developed countries such as the United States, the most commonly reported unused medications were treatments for chronic conditions such as diabetes, hypertension, hyperlipidemia, heart disease, and antipsychotic agents (Law et al., 2015). Meanwhile, analgesics, antibiotics, and herbal medicines were the most reported unused medications in developing countries, such as Indonesia and Nigeria (Auta et al., 2011; Insani et al., 2020).

Improper disposal of unused medications has been reported as a global issue (Tong et al., 2011; Paut Kusturica et al., 2017). The reasons underlying improper disposal of unused medications are lack of policies for returning unused medications and lack of awareness of the impacts of improper medication disposal, such as higher healthcare costs and environmental harm (Wasserfallen et al., 2003; Tong et al., 2011; Bekker et al., 2019; Makki et al., 2019; Insani et al., 2020). For example, a study in the United States showed that improperly disposed of antibiotics may lead to drug-resistant bacteria in soil that can then infect humans (Ghosh and LaPara, 2007). Furthermore, previous studies in France and Pakistan showed adverse effects of improperly disposed of pharmaceutical waste in fish and vultures (Oaks et al., 2004; Sanchez et al., 2011). The environmental impact of improper medication disposal is expected to be higher in countries with poorly functioning waste management schemes such as the Middle Eastern, Asian, and African countries (Paut Kusturica et al., 2017). In a previous study among 497 respondents in Indonesia, most people disposed of unused medications in their household garbage (82.1%), whereas only less than 1% returned unused medications to pharmacies (Insani et al., 2020). Therefore, efforts to reduce improper medication disposal practices are urgently needed.

In 2015, the Indonesian government initiated a community empowerment program called the “Smart Use of Medication Movement” (*Gerakan Masyarakat Cerdas Menggunakan Obat*)*,* which promoted the rational use of medications, including a proper disposal practice of unused medications (Hermansyah et al., 2020). This program encourages community pharmacists to provide general education on how to obtain, use, and store medications and dispose of unused medications. In particular, unused medication disposal should follow predefined regulations to prevent being retrieved or reused prior to destruction (Hermansyah et al., 2018). Nevertheless, nationwide implementation of this program is lacking.

The issue of medication disposal practices among the general population in Indonesia has not been studied comprehensively, despite its potential importance. To strengthen the pharmaceutical waste management program in Indonesia, insights are needed into the association between sociodemographic and other related factors and a lack of awareness of the impact of improperly disposed of unused medications. Therefore, the primary objective of this study was to identify factors associated with a lack of awareness of the impact of improperly disposed of medications among the general population in Bandung, Indonesia. The secondary objective was to assess the associations of awareness with medication disposal practices among this population.

## Patients and Methods

We reported our study according to the Strengthening the Reporting of Observational Studies in Epidemiology (STROBE) guidelines for a cross-sectional study (Von Elm et al., 2007; Supplementary Table S1; Supplementary data).

### Study Design, Setting, and Data Collection

An observational cross-sectional survey was conducted in Bandung, Indonesia, from November 2017 to January 2018. Bandung is the administrative capital of the West Java province and the third most populous city in Indonesia after Jakarta and Surabaya (Tarigan et al., 2016). Data were collected from respondents who met the following inclusion criteria: older than 18 years, used any medication in the past, literate, and signed an informed consent document; we excluded respondents with incomplete data. The Health Research Ethics Committee of Universitas Padjadjaran, Indonesia approved the study protocol (No. 1155/UN6. C.10/PN/2017).

We used a structured questionnaire in Bahasa Indonesia which was developed based on theoretical frameworks of behavior used in previous studies (West et al., 2016; Bashaar et al., 2017). We tested the questionnaire’s validity and reliability with 20 respondents who were not familiar with the study prior to the actual data collection to assess the applicability and clarity of the questionnaire as well as to make necessary adjustments. These respondents were excluded in the main analysis. We made minor revisions to some of the wording and the final version of the questionnaire showed adequate validity (correlation of each question to the total score >0.349) and reliability (Cronbach’s α = 0.854).

Data were collected by either an online- or a paper-based questionnaire using nonprobability sampling. The online-based questionnaire was hosted online using Google form and the link was distributed through social media. Meanwhile, the paper-based questionnaires were distributed to respondents in public places such as city center, markets, and religious places. The questionnaire consisted of two sections: respondents’ sociodemographic characteristics (age, gender, highest education level, occupation, and income) and information on unused medication disposal (e.g., what respondents did with unused medications, whether respondents have ever received information about proper medication disposal practices, the number of medications stored at home, and awareness regarding medication disposal practices) (Insani et al., 2020). Unused medications refer to deteriorated, discontinued, expired, and other medications unintended for future use (Yimenu et al., 2020). The term “medication” encompassed prescribed medications, over-the-counter medications, supplements, vitamins, and herbal medicines. We measured respondents’ awareness regarding medication disposal practices by asking the following question: “Are you aware that improper medication disposal could harm the environment and population health? (yes/no)” (Bashaar et al., 2017).

### Sample Size Calculation

We calculated the sample size using Slovin’s formula (Almeda et al., 2010). To obtain a 95% confidence interval (CI) and a margin error of 0.05, a minimum sample size of 400 respondents was needed.

### Data Analysis

We used descriptive statistics to report respondents’ characteristics and performed chi-square tests to assess univariate associations of dichotomous or nominal factors with their lack of awareness. We included the potential factors that were associated with a lack of awareness in the univariate analyses at a significance of *p* < 0.25 in the initial multivariate models and tested for multicollinearity to check the correlations among the potential factors. Using manual backward elimination, we obtained odds ratios (ORs) with 95% CIs using binary logistic regression analysis. We checked the completeness, accuracy, and consistency of the collected data before entering the data into the statistical software, SPSS version 23.0 (IBM Corp. Armonk, NY, United States).

## Results

### Baseline Characteristics

**FIGURE 1:**
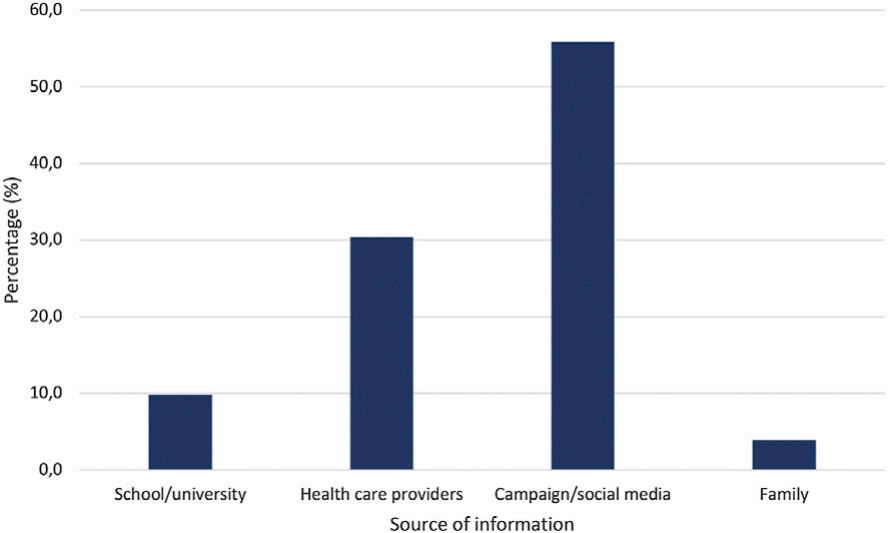
Source of information about medication disposal practice.

Of 497 participating respondents, 433 and 64 respondents filled an online- or a paper-based questionnaire, respectively. Most respondents were female, aged between 18 and 30 years, and students/university students (Table 1). More than half of respondents (53.1%) reported lacking awareness of proper medication disposal practices, and less than 21% of respondents had ever received information about proper medication disposal (Table 1). Among those who had received such information, the most common source had been public campaigns or social media (55.9%), followed by information from health care providers (30.4%; Figure 1).

**TABLE 1:** Demographic characteristics of the respondents (N = 497).

Characteristic	N (%)
Gender	
Male	131 (26.4)
Female	366 (73.6)
Age, years	
18–30	424 (85.3)
31–40	19 (3.8)
41–49	38 (7.7)
50–59	16 (3.2)
Last education level	
Primary school	4 (0.8)
Junior high school	10 (2.0)
Senior high school	316 (63.6)
Diploma/bachelor’s degree	150 (30.2)
Postgraduate degree	17 (3.4)
Occupation	
Students/university students	343 (69.0)
Employed	115 (23.1)
Unemployed	39 (7.8)
Income, Indonesian rupiah	
<1,000,000	229 (46.1)
1,000,000–3,000,000	180 (36.2)
3,000,000–5,000,000	43 (8.7)
>5,000,0000	45 (9.0)
Number of medications stored at home	
None	22 (4.4)
1–5	327 (65.8)
6–10	84 (16.9)
>10	64 (12.9)

### Factors Associated With a Lack of Awareness of Proper Medication Disposal Practices

Results from the multicollinearity test showed that no factors correlated highly with each other (variance inflation factors >1). From the univariate analysis, education, the number of medications stored at home, and previous education about medication disposal practices were the potential factors that were associated with a lack of awareness (Table 2). In the final multivariate model, only those with previous education about medication disposal practices were less likely to report a lack of awareness (OR: 0.043; 95% CI: 0.02–0.09; Table 3). For the goodness of fit of the final multivariate model, *p* was 0.362 and *R*-squared was 23.6% (Table 3).

**TABLE 2:** Univariate association with lack of awareness of proper medication disposal practices (N = 497).

Factor	Aware that improper medication disposal could harm the environment and population health		
	Yes, N (%)	No, N (%)	*p*
Gender (N)			0.368
Male (131)	57 (24.5)	74 (28.0)	
Female (366)	176 (75.5)	190 (72.0)	
Age-group, years (N)			
18–30 (424)	192 (82.4)	232 (87.9)	0.351
31–40 (19)	11 (4.7)	8 (3.0)	
41–49 (38)	22 (9.4)	16 (6.1)	
50–59 (16)	8 (3.4)	8 (3.0)	
Highest education level (N)			0.053*
Primary school (4)	4 (1.7)	0	
Intermediate/secondary school (10)	5 (2.1)	5 (1.9)	
Senior high school (316)	140 (60.1)	176 (66.7)	
Diploma/bachelor’s degree (150)	72 (30.9)	78 (29.5)	
Postgraduate degree (17)	12 (5.2)	5 (1.9)	
Occupation (N)			0.554
Students/university students (343)	156 (67.0)	187 (70.8)	
Employed (115)	56 (24.0)	59 (22.3)	
Unemployed (39)	21 (9.0)	18 (6.8)	
Income, Indonesian rupiah (N)		0.428	
<1,000,000 (229)	112 (48.1)	117 (44.3)	
1,000,000–3,000,000 (180)	76 (32.6)	104 (39.4)	
3,000,000–5,000,000 (43)	23 (9.9)	20 (7.6)	
>5,000,0000 (45)	22 (9.4)	23 (8.7)	
Have received information about medication disposal practices (N)			<0.001*
Yes (102)	94 (40.3)	8 (3.0)	
No (395)	139 (59.7)	256 (97.0)	
Number of medications stored at home (N)			0.187*
None (22)	14 (6.0)	8 (3.0)	
1–5 (327)	147 (63.1)	180 (68.2)	
6–10 (84)	37 (15.9)	47 (17.8)	
>10 (64)	35 (15.0)	29 (11.0)	

* Included into multivariate analysis.

**TABLE 3:** Factor associated with lack of awareness of proper medication disposal practices.

Factor^a^	Odds ratios^b^ (95% CI)
Have received information about medication disposal practices	
Yes	0.043 (0.02–0.09)
No	References

a Goodness of fit: *p* = 0.362, *R*-squared, 23.6%.

b Final multivariate model.

### Associations Between Awareness and Medication Disposal Practices

We observed a significant association between the respondents’ awareness of the impact of improperly disposed of medications and their actual disposal practices. Among respondents who were not aware that disposing of unused medications improperly could harm the environment and population health (N = 264), 56.6% disposed of their unused medications in their household garbage (Table 4). Similarly, respondents who were unaware of the harmful impacts of improper medication disposal shared their unused medications with friends or relatives (53.8%). In contrast, among the respondents who reported being aware of the negative impacts of improper medication disposal (N = 233), 76.9% disposed of their unused medications by flushing them down the toilet or the sink (Table 4).

**TABLE 4:** Associations between awareness and actual unused medication disposal practices.

	Actual practices of disposal of unused medications						*p*
	Threw away in household garbage (N = 408)	Flushed down the toilet or sink (N = 26)	Burned the medications (N = 20)	Shared with friends and/or relatives (N = 286)	Returned it to pharmacy (N = 1)	Did not know (N = 40)	
Aware that improper medication disposal could harm the environment and population health							0.001
Yes (N = 233)	177 (43.4%)	20 (76.9%)	15 (75.5%)	132 (46.2%)	1 (100%)	18 (45.5%)	
No (N = 264)	231 (56.6%)	6 (23.1%)	5 (25.5%)	154 (53.8%)	0	22 (55.0%)	

## Discussion

More than half of the respondents in our study were not aware of the impacts of improper medication disposal on the environment and on population health. Respondents who had previous education on medication disposal practices were less likely to report a lack of awareness, and respondents with a lack of awareness tended to dispose of their unused medications in the garbage or shared them with friends or relatives.

In our study, despite the respondents’ high education levels, they reported a lack of awareness of the impacts of improperly disposed of medications. We found a higher lack of awareness in our study than what previous researchers found in a Swedish population (Persson et al., 2009). Researchers in that previous study reported that information campaigns increased awareness among the Swedish population as indicated by the higher rate of returning medications to pharmacies and the lower rate of discarding them in the garbage (Persson et al., 2009). Our study findings support education as a critical means of raising awareness of the impact of improper medication disposal practices. Therefore, improving the public’s medication disposal practices will require involving healthcare providers in educating the general population about good practices through campaigns and health promotions using various media.

We further observed that gaps exist in medication disposal awareness and practices. Although 46.9% of the respondents in our study were aware that improper medication disposal could harm the environment and population health, a significant number (76.9%) disposed of their unused medications by flushing them down the toilet or sink in their households. This might be because of a misunderstanding that wastewater treatment will remove most of the medications from the environment and ecosystem (Ong et al., 2020). However, it is instead the case that medications disposed of in the public sewage system can contaminate local water systems through groundwater, streams, lakes, and rivers (Jones et al., 2004). Previous studies have reported that proper medication disposal practices are still lacking even among respondents who were aware of the impacts of improper disposal (Sonowal et al., 2017; Ariffin and Zakili, 2019). However, it is still important to reduce the lack of awareness, which can be achieved by engaging respondents with appropriate and accessible information (Bond et al., 2012). In a larger context, the Indonesian government should introduce more nationwide programs such as awareness raising campaigns with various media to educate the general population. In the United States, social media, radio, and television have been used to increase population awareness of the proper disposal of unused opioid pain medications (U.S. Food and Drug Administration (FDA), 2020). Other study in the United States observed that pharmacists receiving the educational intervention were more likely to recommend proper methods of medication disposal during patients’ counseling (Jarvis et al., 2009). Counseled patients were more likely to properly return unused medications to pharmacies and healthcare providers (Seehusen and Edwards, 2006). Moreover, previous studies in the United Kingdom and Sweden showed that information campaigns to increase awareness resulted in a higher rate of returning medications to pharmacies (O’Sullivan et al., 1996; Persson et al., 2009).

To our knowledge, this is the first thorough evaluation of factors associated with lack of public awareness of proper disposal of unused medications and the associations between the individuals’ awareness and their actual medication disposal practices among Indonesian respondents. However, some limitations need to be mentioned. Awareness may overestimate the actual respondents’ knowledge on unused medication disposal practices. In addition, we used a self-report questionnaire which could have introduced recall and social desirability bias and, in turn, led to overestimating respondents’ awareness and their actual disposal practices. We could not verify the information we collected from the respondents, and direct observation such as through in-home inventory of unused medications would allow for objective assessments of unused medication disposal practices. However, this study approach was hindered by resource constraints and privacy concerns. Furthermore, we could not calculate the response rate due to lack of data regarding the number of potentially eligible respondents who refused to participate in this study. We also could not draw causal inferences regarding the temporal associations between previous education and lack of awareness, as well as between lack of awareness and actual medication disposal practices because of the cross-sectional design. The overall association of the final multivariate model was relatively low, suggesting that other unmeasured factors might have been associated with awareness of proper medication disposal practices, such as beliefs about good practices (Seehusen and Edwards, 2006). Furthermore, most of the respondents in our study were university students and thus might not be representative of the general population. Therefore, we advise caution in interpreting and extrapolating our results beyond Bandung city, Indonesia. Finally, generalization of our results may be limited because this was a survey from one location in Indonesia.

Strengthening the current state of unused medication disposal practices in Indonesia will require an organized method for collecting unused medications from the general population and disposing of them properly through practical policies such as pharmacy-based take-back medication programs. Such programs were shown to improve medication disposal practices in developed countries such as the United States (Yang et al., 2015), Australia (Bettington et al., 2018), and the United Kingdom (Mackridge and Marriott, 2007). The availability of such program may alleviate medication misuse and environmental damage associated with improper disposal of unused medications. In addition, efforts should be ongoing to raise awareness through a wide variety of educational interventions to improve medication disposal practices in Indonesia. Healthcare providers could discuss proper medication disposal during counseling and distribute written materials with medications. Future research should focus on qualitative research to provide more in-depth guidance for developing such strategies. Furthermore, a better understanding of the knowledge and attitudes relating to medication disposal of different groups would give a more comprehensive overview of medication disposal practices. Such findings might support research on how to effectively educate the population on proper medication disposal practices.

## Conclusion

Of the 497 respondents to our survey, half reported a lack of awareness of the impacts of improper medication disposal practices. There is a clear need to increase awareness of the importance of proper disposal practices, in particular among the student population of Bandung city, Indonesia. Healthcare providers can play an important role by educating this specific population on the proper disposal of unused medications.

## Data Availability

The raw data supporting the conclusion of this article will be made available by the authors, without undue reservation.
